# Notes on Preparations for the Sick

**Published:** 1896-09

**Authors:** 


### Notes on Preparations for the Sick.

**Glycerole Chloride of Iron; Dialysed Iron; Wine of Tar; Compressed Pills (or Powders).**—John Wyeth & Brother, London. The **Glycerole Chloride of Iron** is non-alcoholic. Each fluid ounce represents sixty drops of tincture of perchloride of iron. The iron exists in the form of a ferric salt. **Dialysed Iron** is a neutral solution of peroxide of iron in a colloid form. This well-known preparation needs no commendation: the colloidal hydrate of iron forms an inodorous liquid of a deep-red colour, but with little styptic taste. These preparations do not give rise to gastric disturbance and do not blacken the teeth.

The **Wine of Tar** is a preparation from an old formula supplied by Dr. Samuel Jackson some thirty years ago; it has long been in use in cases of bronchial and gastric catarrh. We question if the quantity of tar is sufficient for modern requirements in the treatment of phthisis, but in many catarrhal conditions of the various mucous membranes it has a long-established reputation.

The **Compressed Pills** have the ordinary tabloid shape, and some are sugar-coated. They appear to be exceedingly well made, and a long list is provided for almost every possible requirement.

---

**Ferropyrin.**—B. Kühn, London.—This, for which Mr. Kühn is the agent, is a dark-red crystalline powder, readily soluble in cold water; it is a compound of one molecule of ferric chloride with three molecules of antipyrin. It has given good results in chlorotic and anæmic conditions, especially when accompanied by headaches, migraine, gastralgias, and similar neuralgias. The success of this combination of the hæmatinic with the analgesic has fulfilled all expectations. Dr. Cubasch, after four years' trial, speaks very highly of its utility in doses of three to four grains three or four times daily.

---

**Taka-Diastase.**—Parke, Davis & Co., London.—Persons who object to the taste and consistence of malt extracts of the ordinary kind may be glad to have the powdered diastase as prepared for them at Detroit, Mich. It is capable of converting over one hundred times its weight of starch into sugar, and it must therefore be a valuable drug in the treatment of amylaceous dyspepsia. Its name arises from the fact that it is prepared from Taka Koji, which is wheat bran in which a fungus, the Eurotium oryzæ, is grown: the diastase is washed from the bran, and is obtained in a state of considerable purity and concentration.

---

**Malto-Peptone Malt Extract Jelly; Malto-Peptone Malt Extract Jelly, with Cod-Liver Oil.**—The Malto-Peptone Co., Needham Market, Suffolk.—The basis of these preparations is what is called a malto-peptone malt extract, which "(1) is prepared from fine diastasic malt specially made for the purpose; (2) contains the whole value of malted barley, not only a high percentage of its diastase . . . but also the soluble nitrogenous matter and natural salts contained in the malt rootlets, which is extracted from them by a special patented process and designated Malto-Peptone. (3) The addition of Malto-Peptone has a two-fold valuable effect: (*a*) it greatly adds to the nutritive principles of malt extract, the nitrogenous constituents being in a soluble and quickly assimilable condition, and is consequently of much benefit in cases where ordinary nutriment is difficult of retention, or even dangerous to take; (*b*) it wonderfully improves the flavour of the malt extract, which has none of the nauseating sickly taste common to most of these preparations, and which frequently is so highly objectionable to invalids, and to those persons whose ailments necessitate a protracted course of treatment with it. (4) It will keep good in any climate and for any reasonable length of time."

The **Jelly** can easily be cut out of the glass jar with a spoon, and has none of the disadvantages of the ordinary sticky syrup. The **Cod-Liver Oil Preparation** should be taken cut up into pieces the size of a large bean, which can be easily swallowed without leaving the flavour of the oil on the palate.

---

**Vin Regno; "Vivo."**—Liebig's Wine Co., Liverpool.—These excellent food products are well worthy of a more extended reputation. The **Vin Regno** is made by adding the juice of five pounds of beef to one quart of port wine. Extract of malt and quinine are also added, or the preparation may be had without quinine. The fluid is clear and is palatable: it has a specific gravity of about 1060, but it does not contain albumen. The "**Vivo**" is a fluid of high gravity, about 1250, containing much albumen: it has a good aroma and flavour. The granular sediment does not contain any blood-corpuscles or muscle-fibres. A dessert-spoonful in boiling water makes a cup of excellent beef-tea.

The Canadian Extract of Beef prepared by this Company consists of the juice of forty pounds of beef concentrated to one pound of the extract.

---

**L.C.C. Meat Juice.**—THE LIQUOR CARNIS COMPANY, Aston Clinton, Bucks.—This very dark-coloured, opaque, viscid fluid is described by its vendors as a "liquid asset," whilst cooked foods, essences, and extracts are, as it were, "bills to be discounted." It certainly is a very concentrated fluid, as when diluted to four times its bulk its specific gravity is as high as 1060. It precipitates freely with picric acid, the albuminometer tube showing a percentage of .4 per cent. in the diluted fluid, making the very high amount of 1.6 per cent. of albumen in the undiluted juice. It contains a quantity of granular sediment with fragments of cells and a few crystals, but we did not identify any fragments of striped muscle or unbroken red blood-cells. The spectroscope does not show the ordinary hæmoglobin bands. The makers say that the "juice consists of the uncooked juices of British beef, highly concentrated, obtained by hydraulic pressure, excluding the indigestible fibre, and retaining only the nutritious portions of the meat in a form that admits of direct absorption." Our examination of the fluid tends to support the above statement: it is the most concentrated fluid we have met with. It has a beefy aroma and flavour, and it may be taken diluted as a cold drink, or to supply the deficient nourishing qualities of a cup of ordinary home-made hot beef-tea, the flavour of which is thereby improved.

---

**Gaskin's Cocoa; "Viking" Condensed Unsweetened Milk.**—ANDERSON & COLTMAN, London.—The chemical composition of this **Cocoa** is shewn by the following analysis (Granville H. Sharpe, F.C.S.):

| | Per cent. | | Per cent. |
|---|---|---|---|
| Albuminous Matters ... ... | 23.75 | Water ... ... ... ... ... | 4.45 |
| Cocoa Butter ... ... ... ... | 31.26 | Ash ... ... .. .. ... ... | 4.43 |
| Theobromin ... ... ... ... | 1.05 | Other Nutritive Matters ... | 35.06 |

It is manufactured in British Guiana; is pure, retaining the flavour and aroma of the cocoa bean; is very nourishing, as shewn by its composition; is easily digested; and one invalid, whom we know and who likes few things, says that this cocoa is very nice.

The **Milk**, from cows grazing in the Norwegian Highlands, unskimmed and condensed without the addition of sugar or any other substance, looks like cream, and when mixed with two or three parts of water it not only closely resembles but also has the chemical composition of fresh milk. Being sterilised, it will keep for any length of time, and may be kept in readiness so as to be at any time available for children and invalids who require sterilised milk of excellent quality. It differs from the ordinary brands in that it does not contain any admixture of cane sugar or any other preservative. For cooking and all other purposes it may be looked upon practically as concentrated sterilised milk.

---

"**Narissa.**"—G. & G. Stern, London.—The composition of this, which is introduced as a new nourishing food, is shown by the published analysis:

| | Per cent. | | Per cent. |
|---|---|---|---|
| Moisture ... ... ... ... ... ... | 4.94 | Starch .. ... .. ... ... | 20.41 |
| Albumen ... ... ... ... ... ... | 21.43 | Woody Fibre ... ... ... ... | 0.56 |
| Fat ... ... ... ... ... ... ... | 8.22 | Mineral Matter (Phosphates and Phosphoric Acid) ... | 5.02 |
| Dextrin ... ... ... ... ... ... | 27.16 | | |
| Milk Sugar and Cane ... ... | 12.26 | | |

A powder of this composition, containing an admixture of albuminous, saccharine, and fatty elements, and easy of digestion, is deservedly spoken of as a model food. It can be taken as a beverage instead of tea or coffee, or as an excellent improvement on oatmeal porridge. Its nutritive value must be superior to that of the ordinary meat essences and juices, and it is by no means unpalatable.

---

**Pitkeathly; Pitkeathly cum Lithiâ.**—Reid & Donald, Perth.—There appear to be as many princes amongst table waters as in the German Empire. This particular "prince," as its proprietors describe it, has many good things said of it, and no one ventures to say anything against it. The water contains the chlorides of sodium, magnesium, and calcium in solution with carbonate of soda. The Lithia aerated water has given much satisfaction in the treatment of gouty affections: each half-bottle contains five grains of the carbonate of lithia.

---

**Australian Brandy.**—Joshua Brothers, London.—It is only natural that the steady development of the Victorian vineyards should lead to the introduction of an Australian genuine grape brandy at a time when the shipments from the Cognac district have so much fallen off. The sample forwarded to us appears to be pure, and certainly its flavour is excellent. Its quality, its price, and the innate love which English people have for things which are not foreign should enable this fluid to compete successfully with Scotch and Irish whisky.

---

**Anæsthetic Tabloids.**—Burroughs, Wellcome & Co., London.—These tabloids are supplied of three degrees of strength, designated "strong," "normal," and "weak." They contain a little morphia and sodium chloride in addition to cocaine, and are intended for hypodermic use in the manner recommended by Dr. C. L. Schleich, of Berlin. We have tried them for removal of a piece of glass from the hand and found them most effectual.

---

**Adeps Lanæ, N.W.K.**—THOMAS CHRISTY & Co., London.—The special features which distinguish this from other preparations of wool-fat are said to be its absolute purity, soft consistency, non-stickiness, and its low melting point.

